# Femoral Neck Strain during Maximal Contraction of Isolated Hip-Spanning Muscle Groups

**DOI:** 10.1155/2017/2873789

**Published:** 2017-03-22

**Authors:** Saulo Martelli

**Affiliations:** Medical Device Research Institute, School of Computer Science, Engineering and Mathematics, Flinders University, Adelaide, SA, Australia

## Abstract

The aim of the study was to investigate femoral neck strain during maximal isometric contraction of the hip-spanning muscles. The musculoskeletal and the femur finite-element models from an elderly white woman were taken from earlier studies. The hip-spanning muscles were grouped by function in six hip-spanning muscle groups. The peak hip and knee moments in the model were matched to corresponding published measurements of the hip and knee moments during maximal isometric exercises about the hip and the knee in elderly participants. The femoral neck strain was calculated using full activation of the agonist muscles at fourteen physiological joint angles. The 5% ± 0.8% of the femoral neck volume exceeded the 90th percentile of the strain distribution across the 84 studied scenarios. Hip extensors, flexors, and abductors generated the highest tension in the proximal neck (2727 *με*), tension (986 *με*) and compression (−2818 *με*) in the anterior and posterior neck, and compression (−2069 *με*) in the distal neck, respectively. Hip extensors and flexors generated the highest neck strain per unit of joint moment (63–67 *με*·m·N^−1^) at extreme hip angles. Therefore, femoral neck strain is heterogeneous and muscle contraction and posture dependent.

## 1. Introduction

Muscle forces are associated with net bone formation [1]. Exercise is thus recommended to mitigate bone loss and the associated risk for fragility fractures, arising from either the natural aging process or prolonged disuse. However, randomized controlled trials of exercise interventions have shown a variable hip response to exercise, yet leaving unresolved the exercise type for optimal osteogenic response in the femoral neck [2]. Information about the muscle-specific mechanical stimulus in the proximal femur may help explain the variable bone response to exercise in the femoral neck and ultimately it may help in designing targeted exercise interventions for promoting hip strength [3].

Areal Bone Mineral Density (aBMD) measurements showed a variable bone response in the proximal femur to diverse exercise interventions [4, 5]. For example, Kohrt and coworkers [4] showed that weight-bearing exercises, for example, jogging, cause a 3.5% aBMD increase in the femoral neck and a 6.1% aBMD increase in Ward's triangle while resistance exercises, for example, weight-lifting and rowing, cause a 5.1% aBMD increase in the Ward's triangle but no aBMD changes in the femoral neck. Lohman and coworkers [5] used a complex exercise program including weight-bearing and resistance exercise showing a 2.0% aBMD increase in the trochanteric region but no aBMD changes in the femoral neck and Ward's triangle. Yet the mechanism underpinning the variable and at times contrasted hip bone response to exercise is not fully understood, complicating the design of exercise interventions for optimal osteogenic response in the femoral neck [2]. Emerging evidence suggests that exercise type dependent muscle contractions could be responsible for a variable mechanical stimulus in the femoral neck. For example, Lang and coworkers [3] showed that squat/deadlift and abduction/adduction exercise programs cause an exercise-specific and spatially heterogeneous volumetric Bone Mineral Density (vBMD) change, which was then attributed to the different muscles contraction driving squat/deadlift and abduction/adduction exercises [3].

The femoral neck mechanics depends on muscle force intensity, orientation, and application point, which in turn are defined by muscle activation, architecture, and body posture. For example, the hamstring muscles generate a hip force vector aligned with the direction through the ischial tuberosity and the posterior aspect of the tibial plateau whereas the gluteus medius force pulls the femur from the greater trochanter toward the iliac crest. The former muscles generate a force vector in a quasi-sagittal plane and mainly aligned with the femoral shaft while the latter muscles generate a force vector in a quasi-frontal plane aligned with the femoral neck axis. Body posture influences muscle length, function, and the musculoskeletal alignment, thus modulating the muscle mechanical effect on the surrounding skeleton. Earlier numerical studies of femoral neck strain accounted for the combined effect of the different muscles during activity, showing that femoral strain is exercise type dependent [6–8]. However, no study has investigated the separate effect of isolated muscle contractions on the femoral neck strain. This information may help understand how muscles separately contribute to femoral neck mechanics, fracture behaviour, and response to exercise.

The aim of this study was to test the hypothesis that the hip-spanning muscles can generate a muscle- and posture-specific strain distribution in the proximal femur. To this purpose, femoral neck strain patterns during maximal isometric contractions of the hip-spanning muscles were calculated over a physiological range of motion for an average Caucasian elderly woman. Principal tensile and compressive strain maps were compared in terms of magnitude and regional distribution, which are known to determine both amount and spatial location of bone mechanoadaptation [1].

## 2. Materials and Methods

Femoral neck strains were calculated using (a) validated musculoskeletal and finite-element models of an average-sized Caucasian woman and (b) hip and knee moment measured during maximal isometric exercises from two cohorts of elderly healthy participants [9, 10]. Contractions of the hip-spanning muscles across a physiological range of hip and knee angles were simulated. The muscle contribution to the hip force vector, femoral neck strain intensity, and spatial distribution were calculated for a range of hip and knee angles within a physiological range likely achievable by healthy adult individuals.

The lower-limb musculoskeletal model and the finite-element model of the right femur were taken from earlier studies [11, 12]. In summary, models were based on an 81-year-old Caucasian woman, 167 cm height and 63 kg mass with no history of musculoskeletal disease. The musculoskeletal model was a 13-segment, 15-degree-of-freedom articulated system, actuated by 84 Hill-type muscle-tendon units. The model of each muscle was identified using the muscles' physiological cross section area (PCSA) extracted from clinical images and a nominal peak muscle tension of 1 MPa. The tendon slack length, optimal fibre length, and pennation angles were adjusted from the work of Delp and coworkers [13]. The hip and knee reference pose was defined by assuming zero hip and knee angles with the donor lying supine. Positive rotation of hip and knee angles was assumed hip abduction, flexion, and knee flexion. The femur was modelled using a locally isotropic ten-node tetrahedron mesh whose geometry and material property distribution were extracted from computer-tomography images. The femur model was previously validated against measurements of cortical strains (*R*^2^ = 0.95; RMSE = 12.5%; maximum error = 35.1%; [6]), while the musculoskeletal model was shown to yield joint angles, moments, hip, and muscle force patterns in agreement with published patterns for a variety of daily activity types [11].

The hip and knee moment generating capacity in the model was matched to that in the elderly population. The peak isometric moment about the hip and the knee measured in two cohorts of healthy controls was taken from the work of Steinhilber et al. [10] and Tan et al. [9] (Table 1). One cohort (five males and eleven females aged 54 to 73) executed maximal isometric exercises about the hip [10] while the second cohort (thirty females of 50.6 ± 9.7 year of age) executed maximal exercises about the knee [9]. The muscles in the model were grouped by function (Table 2). The peak isometric moment measured for each exercise (Table 1) was associated with the corresponding agonist muscle group (Table 2), assuming no antagonist muscle contraction. The muscle contraction velocity was set to zero (isometric) and the muscle activation was set to 1 (maximal effort). For each exercise and muscle group *j*, the peak isometric moment *Mm*(∝)^*j*^ in the model was calculated using the equation

(1) $$\begin{array}{c} {Mm\left(\propto \right)}^{j}=\underset{i}{\sum }{f\left(\propto \right)}_{i}^{j}\times {l\left(\propto \right)}_{i}^{j}, \end{array}$$

where ∝ is the joint angle; *f*(∝)_*i*_^*j*^ is the peak isometric muscle force of the muscle *i*; and *l*(∝)_*i*_^*j*^ is the corresponding muscle lever arm. The peak isometric muscle force *f*(∝)_*j*_^*i*^ was then scaled using the equation

(2) $$\begin{array}{c} {f\left(\propto \right)}_{j}^{i}=\frac{{Me\left(\propto \right)}^{j}}{{Mm\left(\propto \right)}^{j}}\times {f\left(\propto \right)}_{j}^{i}, \end{array}$$

where *Me*(∝)^*j*^ is the average moment measured in the elderly population. For the maximal knee extension and flexion exercises, the knee angle (∝) was set to 60° and 30° according to the work of Tan et al. [9] (Table 1) while for the maximal exercises about the hip the joint angle ∝ was set to the hip angle at the peak isometric moment in the model. The hip and knee moment and the corresponding muscle forces in the model are reported in the Supplementary Figures  1 and 2 (see Supplementary Material available online at https://doi.org/10.1155/2017/2873789).

**Figure 1:**
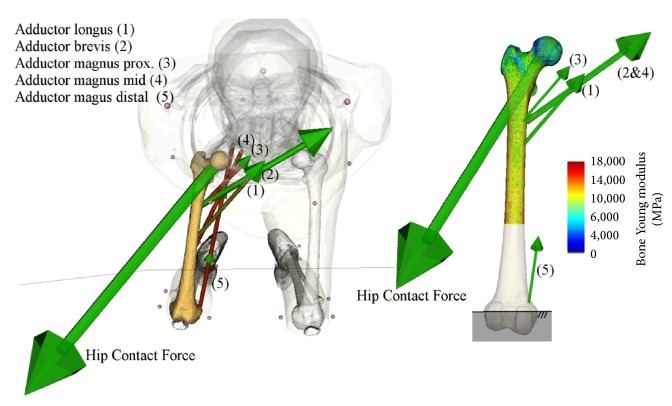
The simulation of isometric contraction of the isolated hip adductor muscles at 0° hip adduction (left) and the linked finite-element model of the femur (right). The femoral young modulus map, muscle, and joint forces are also displayed.

For each exercise and agonist muscle group, femoral strains were calculated using the peak isometric force of the hip-spanning muscles for 14 intermediate joint angles uniformly distributed within a physiological range: hip abduction angle ranged between −39° and 0°, the hip flexion angle ranged between −20° and 30°, and knee flexion angles ranged between 0° and 90° (Table 2). The hip contact force was calculated by solving for static equilibrium. The finite-element model of the femur was distally fully constrained. Muscle and hip contact forces were applied to the finite-element model using an in-house routine (Matlab©, The Mathworks, USA) (Figure 1). Principal compressive and tensile strains were calculated using the direct linear-elastic solver implemented in Abaqus© (Dassault Systemes, USA) resulting in 84 tensile and compressive strain maps (6 muscle groups, 14 joint angles). Tensile and compressive strain over the femoral neck cortex were averaged over a 3 mm diameter region. This procedure was shown to produce a reliable representation of femoral strain and fracture load [14].

The musculoskeletal anthropometry, the femoral geometry, and mineral content in the model were compared to corresponding values in elderly Caucasian women. The model height and weight were compared to corresponding values from the Centers for Disease Control and Prevention (US Department of Health and Human Services, https://www.cdc.gov/). Muscle moment arms were compared to corresponding published values obtained using different procedures [15–18]. The femoral neck diameter, femoral neck length, anteversion angle, and craniocaudal angle were compared with the literature [19–21]. The aBMD in the femur was extracted from the CT data following the guidelines by Khoo et al. [22] and compared to corresponding values in a cohort of elderly Caucasian women [23].

The hip force vector was expressed in the femoral coordinate system and compared with measurements taken from implanted patients during walking, stair ascent, and stair descent by Bergmann et al. [24].

The calculated strain maps were compared to provide a relative information about the potential of the different muscle groups for generating localised tensile and compressive strain over the femoral neck. The heterogeneity of the strain map was assessed by calculating the neck volume and cortex area exceeding the 75th and 90th percentile of the strain distribution. The relative capacity of each muscle group to load the femoral neck was assessed by comparing the peak and the average tensile and compressive strain over the studied joint angles.

The effect of different hip and knee joint angles on femoral neck strain was assessed by calculating the range of the peak tensile and compressive strain generated by each muscle group across the studied joint angles and by normalizing the calculated peak strain by the corresponding joint moment. The peak strain range provides information about the combined effect of the different geometrical musculoskeletal arrangement and muscle force generating capacity induced by changes of body posture. The normalized peak strain provided the distinct effect of the different geometrical musculoskeletal arrangement on neck strain and it represents the specific neck strain generating capacity per unit joint moment.

## 3. Results

The model represented an average elderly Caucasian woman. The height and weight of the model (167 cm height; 63 kg weight) compared well with the average Caucasian woman 60 years of age and over (173.8 cm height; 71.9 kg weight; [25]). The femoral length, head diameter, femoral neck length, and the craniocaudal angle were within one standard deviation (*Z*-score < 1) from corresponding average values in the same population while the *Z*-score for the femoral anteversion angle was 1.1 [19–21] (Supplementary Table  1). The aBMD was 0.53 g·cm^−2^ (*T*-score = −2.5), which represents the 30th percentile of elderly Caucasian women [23]. The range of muscle moment arms over the studied joint angles compared well with earlier MRI studies [16, 26], an in vivo noninvasive protocol [17], and dissection studies [18, 27] (Table 3).

**Figure 2:**
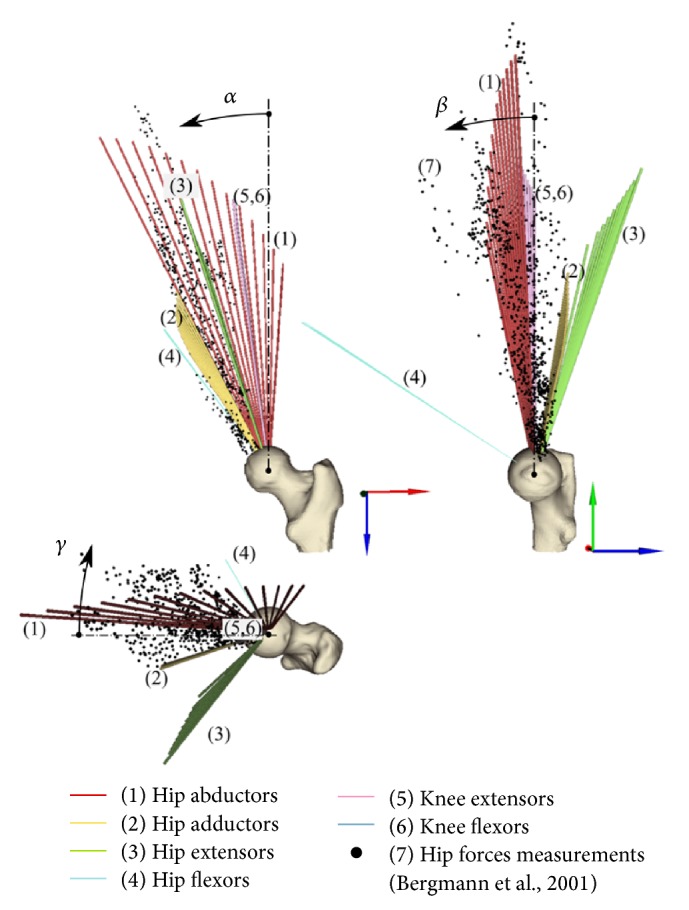
The calculated hip force vector generated by peak isometric contraction of the hip-spanning muscles. The femoral coordinate system is displayed in the posterior and medial views. The hip contact force measured by Bergmann et al. (2001) on three patients (i.e., SNK, KWR, and PFL) during a variety of daily activity is displayed as black dots. Reference zero and positive angle rotations in the frontal (*α*), sagittal (*β*), and transversal (*γ*) planes are also displayed.

The calculated hip contact force ranged from 223 N to 2171 N during isometric contraction of the knee extensor and hip abductor muscles, respectively (Supplementary Figure  3). The hip abductor and adductor muscles generated a hip force that mainly lay in the frontal plane; the former was oriented from 27° to −4° while the latter was oriented from 34° to 27° as the hip adduction angle changed between 0° and −39° hip adduction. The hip extensor and flexor muscles generated a hip force that mainly lay in the sagittal plane; the former oriented from −11° to −17° while the latter was constantly oriented at 53° as the hip flexion angle changed between 30° and −20° hip flexion. Knee flexor and extensor muscles similarly generated a hip force vector within 8° from the femoral shaft axis and changed as little as 4° as the knee flexion angle changed between 0° and 90°. The orientation of the hip force vector measured by Bergmann et al. [24] over different motor tasks and subjects was within and narrower than the calculated range of the hip force vector orientation (Figure 2).

**Figure 3:**
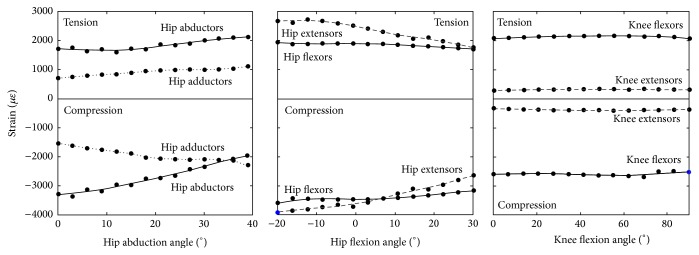
The peak tensile and compressive strain generated by isometric contractions of the hip-spanning muscles across the investigated physiological joint angles.

**Figure 4:**
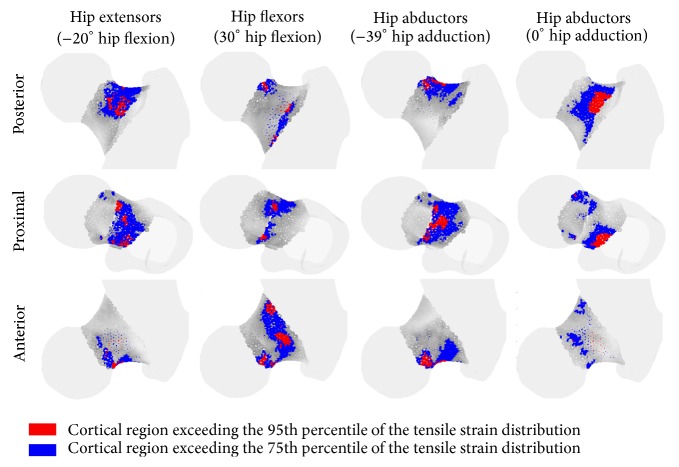
The tensile strain distribution in the femoral neck for selected loading conditions. Posterior, proximal, and anterior views are displayed. The blue and red regions represent the neck cortex region subjected to tensile strain levels above the 75th and 95th percentile of the tensile strain distribution.

The proximal neck was always mainly in tension while the distal neck was always mainly in compression. The femoral neck volume exceeding the 90th percentile of the tensile and compressive strain distribution across the studied muscle contractions and joint angles were the 5% ± 0.8% of the total femoral neck volume. The peak tensile strain ranged between 2727 *με* and 276 *με* while the peak compressive strain ranged between −3907 *με* and −324 *με*. Hip extensor and knee extensor contraction generated the highest strain values (Figure 3). The tensile strain map was variable showing a muscle- and posture-dependent distribution (Figure 4) while the compressive strain map was found less variable and less dependent on exercise type and postural changes.

**Figure 5:**
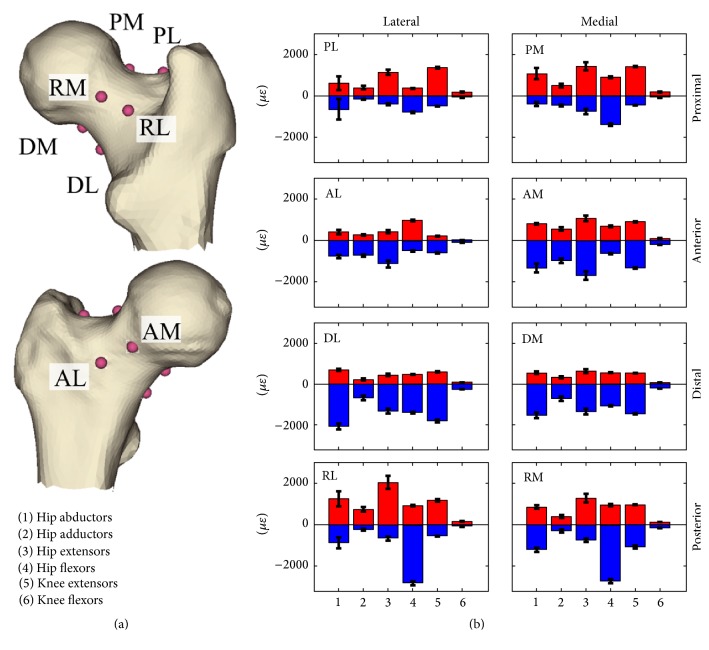
The monitored eight locations (a) of the femoral neck cortex: proximal-lateral (PL), proximal-medial (PM), posterior-lateral (RL), posterior-medial (PM), distal-lateral (DL), distal-medial (DM), anterior-lateral (AL), and anterior-medial (AM). The boxplot (b) represents the average tensile (red) and compressive (blue) strain at the monitored locations during contractions of the hip-spanning muscles (1–6). The error bar represents ± standard deviation.

In average terms, hip extensors contraction induced the highest tensile strain over the studied hip flexion angles, reaching the highest tensile strain (1433 *με*) in the proximal-medial neck and the highest compressive strain (−1671 *με*) in the anterior-medial neck. Hip flexor muscle contraction induced the highest tensile strain (986 *με*), averaged across the studied hip flexion angles, in the anterior-lateral neck and the highest compressive strain (−2818 *με*) in the posterior-lateral neck. Hip abductor muscles induced the highest compressive strain in the distal-lateral neck (−2069 *με*) (Figure 5).

**Figure 6:**
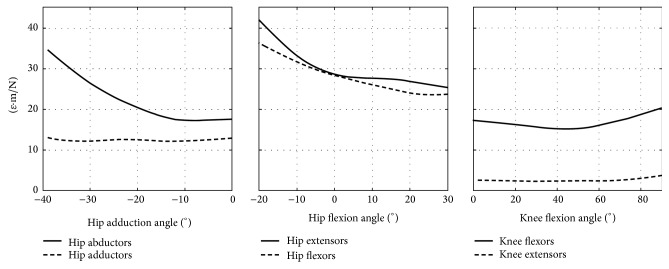
The normalized peak tensile strain across the studied hip-spanning muscles and joint angles.

Variations of hip and knee angle induced a variable effect on femoral neck strain across the different muscle group and joint angles. The peak tensile strain in the femoral neck cortex changed by 974 *με* (36% of the peak tensile strain) during hip extensor contraction as the hip flexion angle varied from 30° to −20° while the peak compressive strain changed by 1386 *με* (41% of the peak compressive strain) during the hip abductor contraction as the hip adduction angle varied from 0° to −39°. Little changes of the femoral neck strain were observed during knee extension and flexion exercises (Figure 3). The normalized tensile and compressive strain were variable across the different exercises. The hip extensor and flexor muscles generated the highest normalized strain averaged over the studied hip flexion angles (30 *με*·m·N^−1^ in tension and 52 *με*·m·N^−1^ in compression) and increased at extreme hip flexion angles reaching 42 *με*·m·N^−1^ in tension and 67 *με*·m·N^−1^ in compression. The hip abductor muscles induced a normalized tensile strain of 22 *με*·m·N^−1^ at 0° hip adduction increasing up to 34 *με*·m·N^−1^ at −39° hip adduction (Figure 6) and a moderately variable peak compressive strain (32 ± 1.2 *με*·m·N^−1^). The hip adductors, knee extensors, and flexors showed a normalized strain lower than 24 *με*·m·N^−1^ and little variation due to postural changes (normalized strain range: <6 *με*·m·N^−1^).

## 4. Discussion

The aim of the study was to assess femoral neck strains in response to maximal isometric contractions of isolated hip-spanning muscles across a physiological range of hip and knee joint angles. The model anatomy and femoral elasticity represented an average-sized osteoporotic Caucasian woman. Simulations mimicked maximal isometric contractions of the principal hip-spanning muscle groups across a physiological range of hip and knee angles in the elderly population. Femoral neck strain was highly localised for each studied scenario while the strain level and location were muscle- and posture-specific.

The calculated 84 femoral strain distributions were highly localised for each investigated scenario: 5% ± 0.8% of the femoral neck volume was subjected to a strain level higher than the 90th percentile of the strain distribution while the peak strain value and location were muscle- and posture-dependant (Figures 4 and 5). In fact, the hip extensor and knee flexor muscles, which have the same thigh muscles in common (Table 2), induced the highest tensile strain in the proximal-posterior neck cortex (1433 *με*) and compressive strain in the anterior neck (−1671 *με*), the hip abductor muscles induced the highest compressive strain in the distal neck (−3353 *με*), and the hip flexor muscles induced the highest compressive strain in the proximal-posterior (−2718 *με*) neck cortex and tensile strain in the anterior neck (1089 *με*). The knee extensor muscles had the lowest potential to load the femoral neck due to the relatively low hip reaction force generated by the rectus femoris (Supplementary Figure  3), which is the only hip-spanning muscle within knee extensor muscle group. The effect of body posture, which can influence the femoral neck strain by altering either the muscle force generating capacity or the geometrical musculoskeletal arrangement (Figure 2), was exercise-specific. In fact, the compressive strain varied during hip abduction contraction in terms of both peak (41% variation of the peak compressive strain) and location (Figure 4) as the hip adduction angle changed while moderate changes of both the peak and location of the peak strain were observed during knee flexion and extension exercises (Figure 3). This observed exercise-specific femoral neck strain was attributable to a combination of muscle architecture, body posture, and muscle force generating properties. The muscle architecture induced a muscle-specific potential for generating femoral neck strain per unit of joint moment and the effect of postural changes was much higher for hip extensors, flexors, and abductors than that observed for the hip adductors, knee flexors, and extensors (Figure 6). As such, for these latter muscles the variable capacity of generating femoral neck strain at different joint angles was mainly attributable to changes of the muscle force generating capacity at different joint angles.

The present study provides a theoretical foundation for explaining the variable mechanoresponse to exercise observed in the femoral neck and for supporting the design of novel exercise intervention for bone health. The model previously demonstrated a valid tool for studying femoral strains during activity in that it was validated against experimental measurements of cortical strain (*R*^2^ = 0.95; [6]) and it provided realistic hip contact force patterns during activity [6, 11]. The general finding of a localised muscle- and posture-specific femoral neck mechanics is in agreement with the heterogeneous and exercise-depended bone response following squat/deadlift and abduction/adduction exercises observed by Lang and coworkers [3]. In the same study, Lang and coworkers [3] also observed a concomitant increase of both the femoral neck bone and the hip extensor muscle strength during squat/deadlift exercise, which is in agreement with the high potential of the hip extensor muscles for loading the neck cortex found in the present study. Another finding of the present study resides in the different normalized strain generated by the different muscle groups (Figure 6). This finding provides a meaning for ranking the effect of the hip-spanning muscles on the femoral neck mechanics independently by the variable muscle strength across individuals [9, 10]. Moreover, the observed tendency for higher femoral neck strains toward extreme hip angles (Figure 6) is in agreement with the notion that bone structure is optimized to sustain habitual loads [28] and it suggests that exercising using nonhabitual and extreme body postures may prove beneficial for femoral neck health, although the decreased muscle force generating capacity toward extreme joint angles expectedly limits the potential benefit of exercising at extreme body postures.

This study has some limitations. Firstly, no interaction between joint angles was considered while more complex body postures could have further widened the range of neck strain maps reported in the present study. This may only have strengthened the conclusion of muscle type- and posture-dependent strains in the femoral neck. Secondly, the joint moment measured during maximal exercises may have been generated by activity of both agonist and antagonist muscles, possibly leading to an underestimation of the calculated force of the agonist muscles. Nevertheless, the up to 20% activity of antagonist muscles that can be expected while executing maximal exercises [29] is smaller than the average 28% variation in the measured joint moments [10]. Hence, the agonist muscle forces used in the present study are representative of maximal exercises in healthy elderly individuals. Thirdly, present results were obtained for a single individual representing an average-sized elderly Caucasian woman, while variations in musculoskeletal geometry and bone quality are known to affect femoral neck strain [30]. Nevertheless, the present study provides a first understanding of the mechanical effect in the femoral neck of the hip-spanning muscles in a single representative individual. Further studies are necessary to expose the variation of femoral neck strain within the population of interest. Fourthly, the orientation of the hip force vector calculated during isolated contraction of the hip-spanning muscle groups was larger than that measured during normal activity (Figure 2). However, this apparent discrepancy should be expected because during normal activity the hip force vector is a weighted function of the force vectors generated by the typically complex set of muscles concurrently active. Fifthly, the non-hip-spanning muscles were not included in the model while they directly contribute to the hip force and femoral strain during dynamic activities by means of the dynamic coupling of the musculoskeletal system [31]. However, present results are relevant in that the hip-spanning muscles generate the majority of the hip contact force [31] and femoral neck strain [6] also during normal dynamic activity. Lastly, the present study focused on the femoral neck cortex while the intracortical trabecular network and other anatomical regions such as the femoral trochanter are important to femoral strength and exercise. More research is necessary to extend the present analysis to different anatomical regions.

Despite the above limitations, the present analysis provides the first quantification of the potential of the hip-spanning muscles for loading the femoral neck cortex in a single elderly Caucasian woman. This information can provide a theoretical foundation for understanding the variable, exercise-specific, and heterogeneous femoral neck response to exercise [3], drive the development of novel exercise interventions to promote femoral neck health [32], and provide insights into understanding the mechanism by which the combination of intense muscle contractions and low bone quality may trigger spontaneous neck fractures [33].

## Supplementary Material

Supplementary table 1: Comparison between salient geometrical parameters in the model and in adult Caucasians (average ± standard deviation).
Supplementary figure 1: The hip and knee moment during isolated isometric contraction of the hip-spanning muscle groups across a physiological range of motion.
Supplementary figure 2: The calculated muscles force during isolated isometric contraction of the hip-spanning muscle groups across a physiological range of motion.
Supplementary figure 3: The calculated hip force magnitude during isolated isometric contraction of the hip-spanning muscle groups across a physiological range of motion.
Supplementary figure 4: The calculated tensile strain maps (top view) calculated using the extreme joint angles studied.

## Figures and Tables

**Table 1:** Joint moments (average ± standard deviation) and the corresponding joint angle during maximal isometric contractions of the hip and knee spanning muscles in healthy controls.

Task	Joint angle (°)	Joint moment (Nm ± SD)
Hip abduction	N/A	110.30 ± 24.90^§^
Hip adduction	N/A	85.4 ± 23.8^§^
Hip flexion	N/A	81.6 ± 17.5^§^
Hip extension	N/A	105.1 ± 44.3^§^
Knee extension	60° knee flexion 30° knee flexion	129.10 ± 40.69°
Knee flexion		70.90 ± 14.20°

^§^Steinhilber et al., 2011 (five healthy males and eleven females aged 54 to 73).

°Tan et al., 1995 (thirty healthy females aged 50.56 ± 9.66).

**Table 2:** The muscles grouped according to function.

Hip abductors
Gluteus medius
Gluteus minimus
Tensor fascia latae
Hip adductors
Adductor brevis
Adductor longus
Adductor magnus
Gracilis
Hip flexors
Ileopsoas
Rectus femoris
Sartorius
Hip extensors
Bicep femoris long head
Gluteus maximus
Semimembranosus
Semitendinosus
Knee extensors
Rectus femoris
Vastus Intermedius
Vastus lateralis
Vastus medialis
Knee flexors
Biceps femoris long head
Biceps femoris short head
Semimembranosus
Semitendinosus

**Table 3:** Moment arm range (minimum–maximum) across a physiological range of motion for each of the studied muscle bundles in the model and corresponding published values.

Muscle bundles		Model	Scheys et al., 2008	Arnold et al., 2000	Bonnefoy et al., 2007	Németh and Ohlsén, 1985	Németh and Ohlsén, 1989
Hip abduction	Gluteus medius anterior	4.7–5.7	1.0–4.0				5.4 ± 0.3^*∗*^
	Gluteus medius medial	5.1–5.7	1.8–4.5				
	Gluteus medius posterior	4.1–4.9	0.7–4.8				
	Gluteus minimus anterior	4.1–4.7	0.2–3.8				4.2 ± 0.2^*∗*^
	Gluteus minimus medial	4.2–5.3	1.0–4.0				
	Gluteus minimus posterior	4.3–5.7	0–4.2				
	Tensor fascia latae	4.7–6.8	2.0–6.5				4.6 ± 0.4^*∗*^

Hip adduction	Adductor brevis	5.4–7.5	1.0–6.0				
	Adductor longus	5.3–7.0	2.2–7.7				
	Adductor magnus superior	6.5–8.2	3.0–6.0			2.4–6.0	7.2 ± 0.3^*∗*^
	Adductor magnus medial	6.7–7.7	3.0–6.0				
	Adductor magnus inferior	3.2–6.2	2.2–6.1				
	Gracilis	8.4–9.4	1.0–7.8				

Hip flexion	Ileopsoas	3.5–4.1		1.8–4.1			
	Rectus femoris	2.1–4.2	1.9–5.0				
	Sartorius	2.0–7.3	1.8–7.0				

Hip extension	Biceps femoris long head	2.5–6.0	0.2–6.0				
	Gluteus maximus anterior	4.6–5.3	N/A–4.0			3.1–7.5	
	Gluteus maximus medial	5.3–6.9	N/A–4.5				
	Gluteus maximus posterior	4.8–9.0	N/A–6.2				
	Semimembranosus	1.4–5.4	N/A–5.9	3.1–4.1			
	Semitendinosus	2.2–6.3	N/A–6.0	3.3–5.2			

Knee flexion	Biceps femoris long head	4.1–5.5			2.1–7.1	4.0–7.0	
	Biceps femoris short head	3.0–4.2					
	Semimembranosus	2.9–4.1		3.1–4.1	2.2–5.5		
	Semitendinosus	2.7–4.4		3.3–5.2			

Knee extension	Rectus femoris	2.5–5.2			2.1–5.1		
	Vastus intermedius	2.6–5.3					
	Vastus lateralis	2.5–5.0					
	Vastus medialis	2.4–4.9					

^*∗*^Average ± standard deviation.
