# Supplementary material for: Femoral Neck Strain during Maximal Contraction of Isolated Hip-Spanning Muscle Groups
Source: Comput Math Methods Med. 2017 Mar 22;2017:2873789. doi: 10.1155/2017/2873789 (PMC5381202; doi:10.1155/2017/2873789)

SUPPLEMENTARY MATERIAL

Supplementary table 1 – Comparison between salient geometrical parameters in the model and in adult Caucasians (average ± standard deviation).

	Model	Noble et al. 1988	Michelotti and Clark, 1999	Jackson et al., 2015
Femoral length (cm)	44.7	43.7 ± 3.5		
Femoral head diameter (cm)	4.6	4.6 ± 0.5	5.2 ± 0.4	
Femoral neck length (cm)	5.2		5.7 ± 0.7	
Anteversion angle (°)	18.5			8.2 ± 9.3
Cranio-caudal angle (°)	123.5	124.7 ± 7.4	128.6 ± 6.3	

Supplementary figure 1 – The hip and knee moment during isolated isometric contraction of the hip-spanning muscle groups across a physiological range of motion.

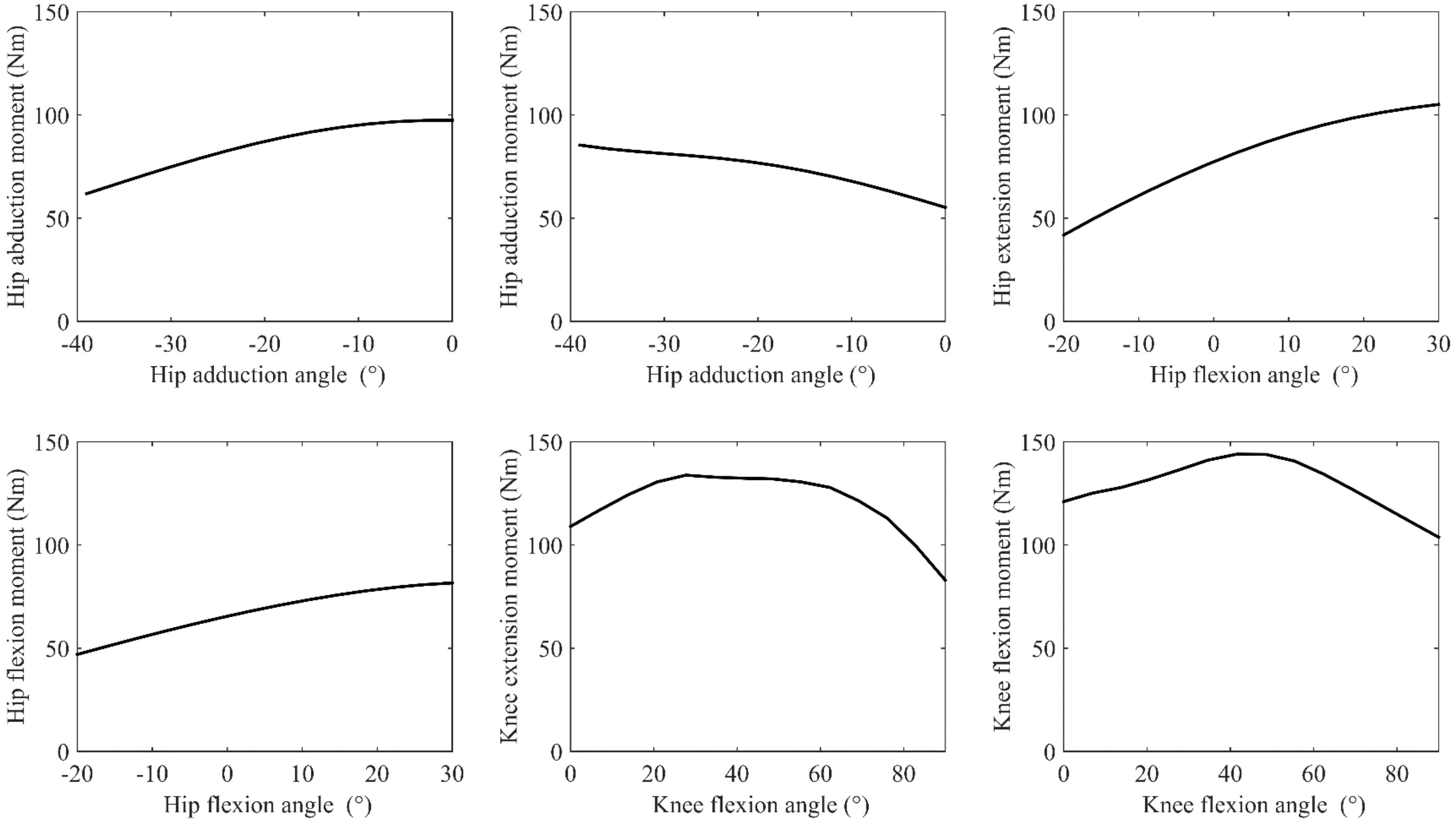

Supplementary figure 2 – The calculated muscles force during isolated isometric contraction of the hip-spanning muscle groups across a physiological range of motion.

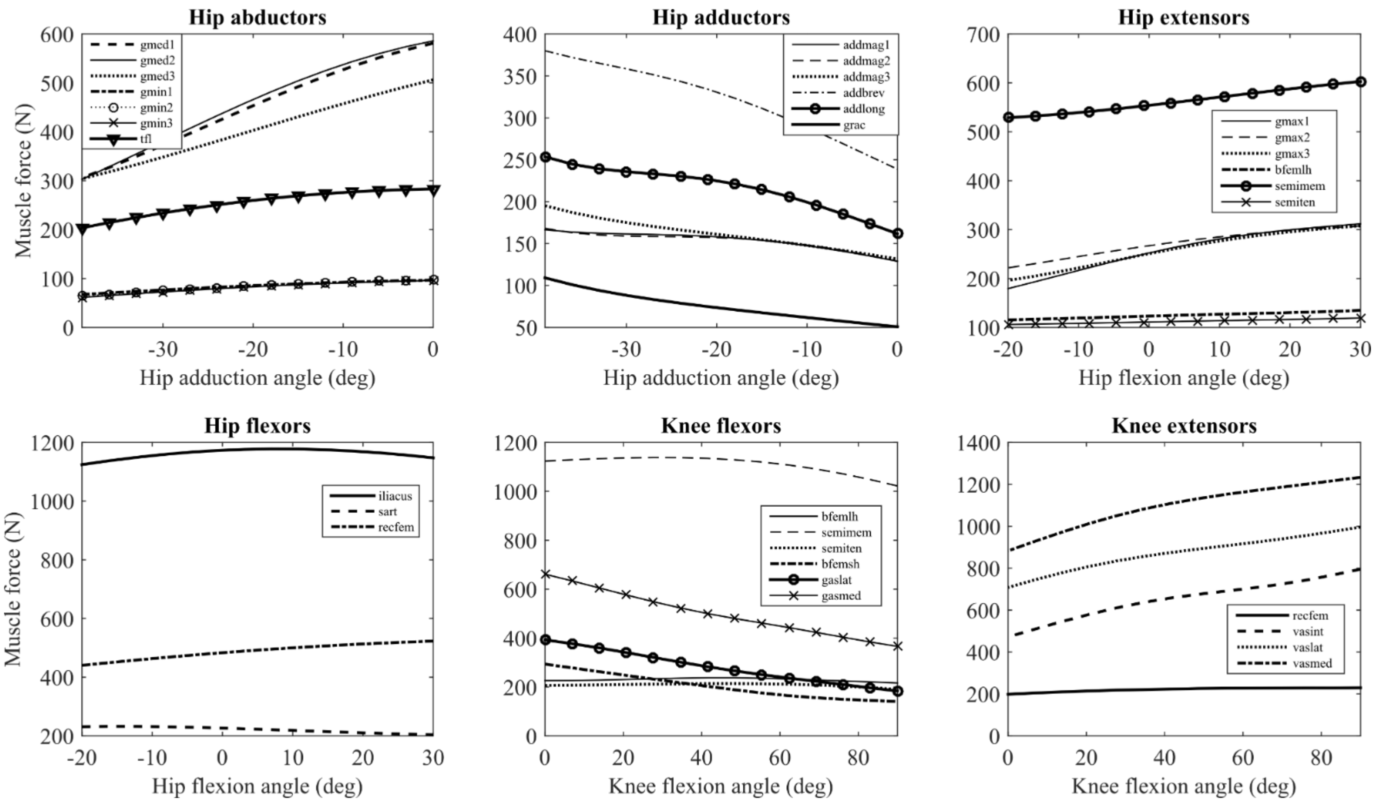

Legend – gluteus medius anterior fibres (gmed1), gluteus medius middle fibres (gmed2), gluteus medius posterior fibres (gmed3), gluteus minimus anterior fibres (gmin1), gluteus minimius middle fibres (gmin2), gluteus minimus posterior fibres (gmin3), tensor fascia latae (tfl), adductor magnus proximal fibres (addmag1), adductor magnus middle fibres (addmag2), adductor magnus distal fibres (addmag3), adductor brevis (addbrev), adductor longus (addlong), gracilis (grac), gluteus maximus anterior fibres (gmax1), gluteus maximus middle fibres (gmax2), gluteus maximus posterior fibres (gmax3), biceps femoris long head (bifemlh), semimebranosus, (semimem), semitendinosus (semiten), iliacus (iliacus), sartorious (sart), rectus femoris (recfem), gastrocnemius lateralis (gaslat), gastrocnemius medialis (gasmed), vastus intermedius (vasint), vastus medialis (vasmed), vastus lateralis (vaslat).

Supplementary figure 3 – The calculated hip force magnitude during isolated isometric contraction of the hip-spanning muscle groups across a physiological range of motion.

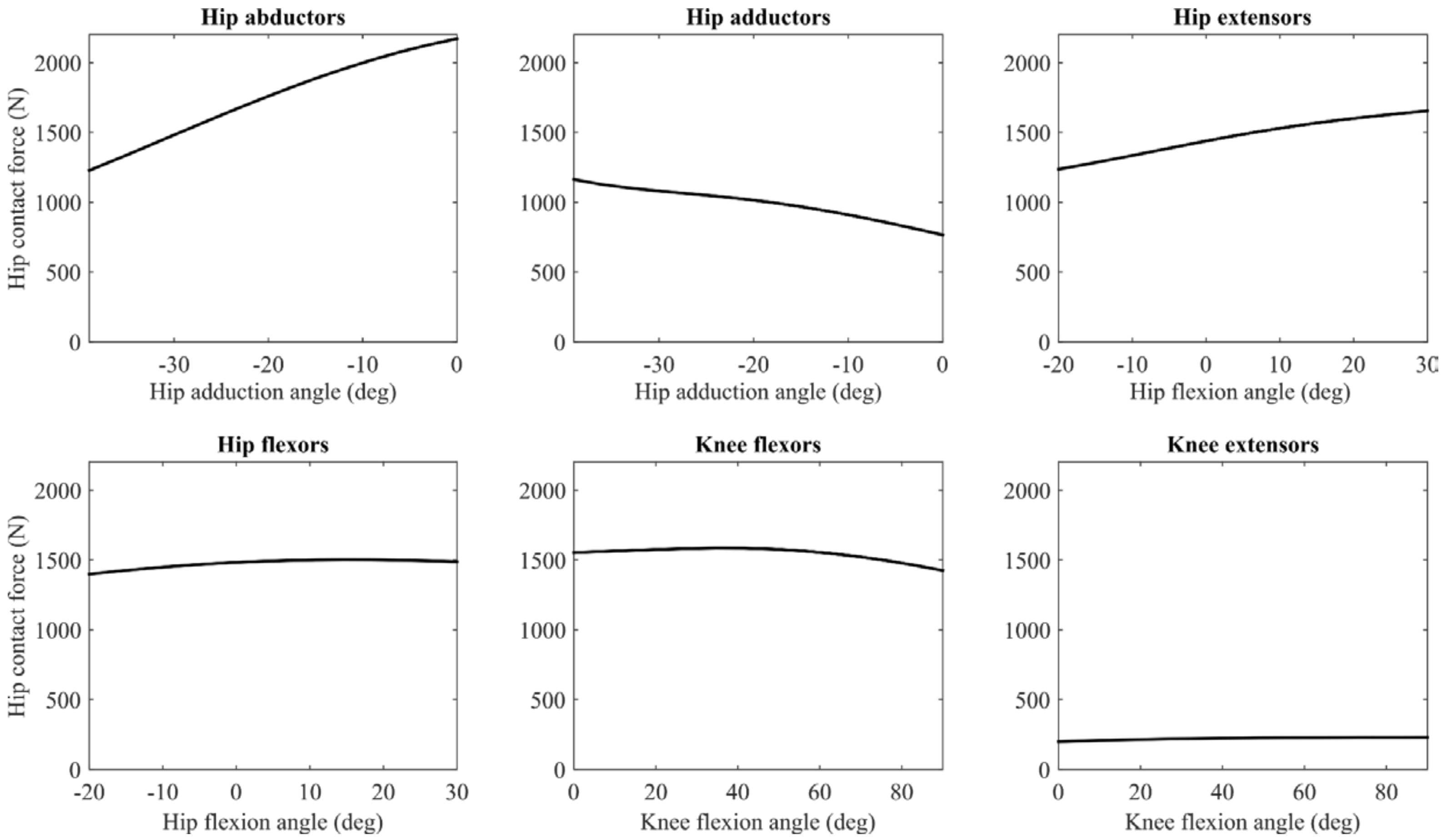

Supplementary figure 4 – The calculated tensile strain maps (top view) calculated using the extreme joint angles studied.

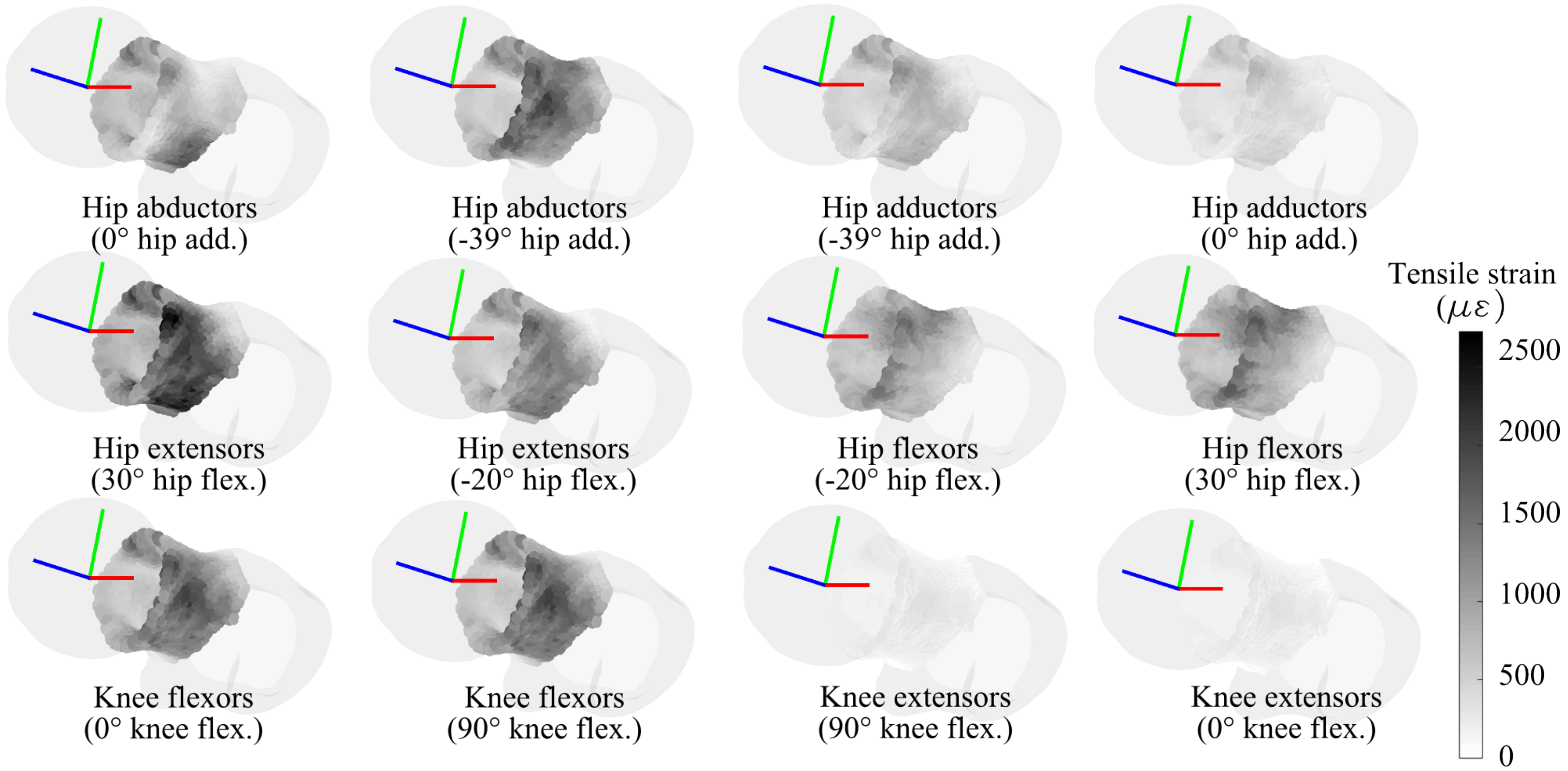

Supplement: Supplementary file 1 — Supplementary table 1: Comparison between salient geometrical parameters in the model and in adult Caucasians (average ± standard deviation). Supplementary figure 1: The hip and knee moment during isolated isometric contraction of the hip-spanning muscle groups across a physiological range of motion. Supplementary figure 2: The calculated muscles force during isolated isometric contraction of the hip-spanning muscle groups across a physiological range of motion. Supplementary figure 3: The calculated hip force magnitude during isolated isometric contraction of the hip-spanning muscle groups across a physiological range of motion. Supplementary figure 4: The calculated tensile strain maps (top view) calculated using the extreme joint angles studied. [file 2873789.f1.zip › martelli2016_supplementary-material_CMMM_1874049.pdf]
