# Supplementary figures and images for: Femoral Neck Strain during Maximal Contraction of Isolated Hip-Spanning Muscle Groups
Source: Comput Math Methods Med. 2017 Mar 22;2017:2873789. doi: 10.1155/2017/2873789 (PMC5381202; doi:10.1155/2017/2873789)

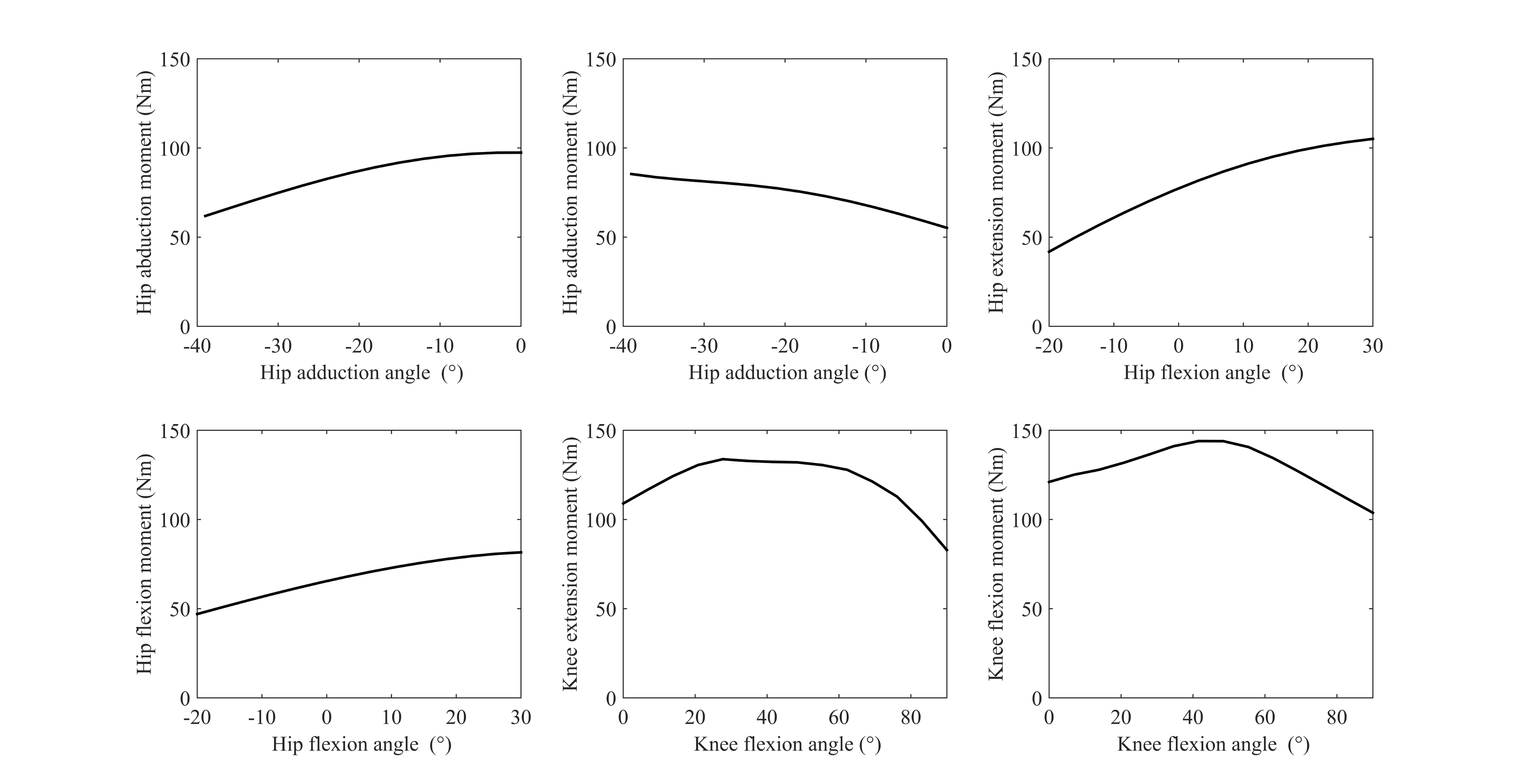

Supplement: Supplementary file 1 — Supplementary table 1: Comparison between salient geometrical parameters in the model and in adult Caucasians (average ± standard deviation). Supplementary figure 1: The hip and knee moment during isolated isometric contraction of the hip-spanning muscle groups across a physiological range of motion. Supplementary figure 2: The calculated muscles force during isolated isometric contraction of the hip-spanning muscle groups across a physiological range of motion. Supplementary figure 3: The calculated hip force magnitude during isolated isometric contraction of the hip-spanning muscle groups across a physiological range of motion. Supplementary figure 4: The calculated tensile strain maps (top view) calculated using the extreme joint angles studied. [file 2873789.f1.zip › suppl_fig1_CMMM_1878443.docx]

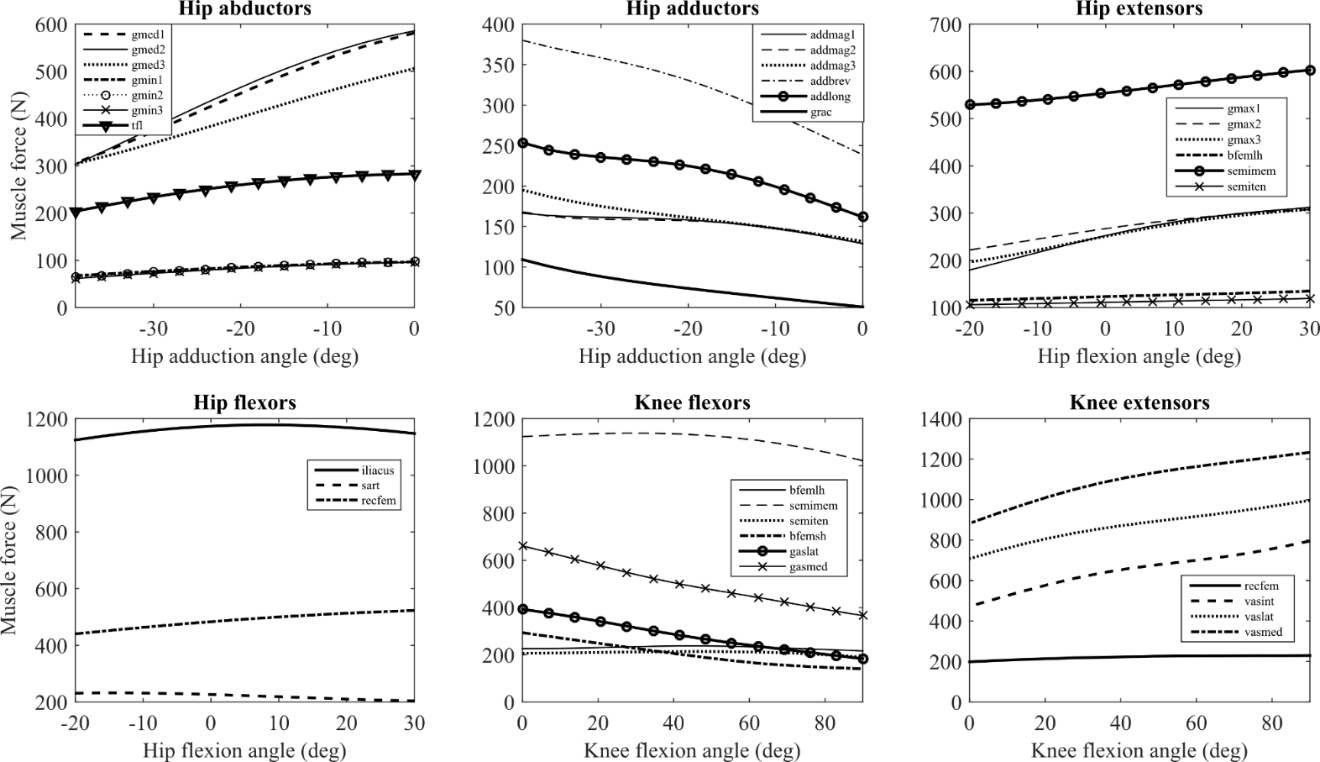

Supplement: Supplementary file 1 — Supplementary table 1: Comparison between salient geometrical parameters in the model and in adult Caucasians (average ± standard deviation). Supplementary figure 1: The hip and knee moment during isolated isometric contraction of the hip-spanning muscle groups across a physiological range of motion. Supplementary figure 2: The calculated muscles force during isolated isometric contraction of the hip-spanning muscle groups across a physiological range of motion. Supplementary figure 3: The calculated hip force magnitude during isolated isometric contraction of the hip-spanning muscle groups across a physiological range of motion. Supplementary figure 4: The calculated tensile strain maps (top view) calculated using the extreme joint angles studied. [file 2873789.f1.zip › suppl_fig2_CMMM_1878444.docx]

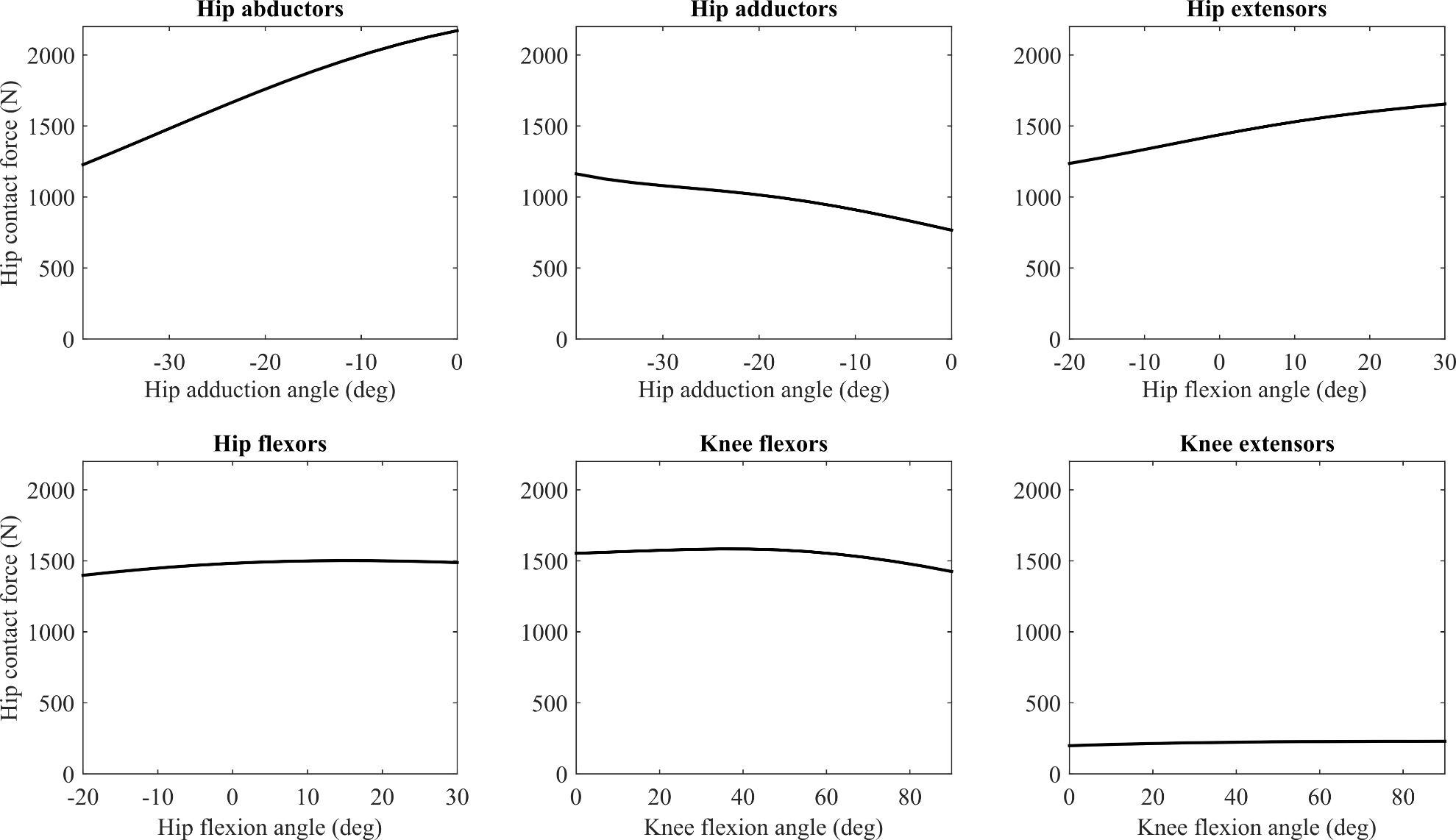

Supplement: Supplementary file 1 — Supplementary table 1: Comparison between salient geometrical parameters in the model and in adult Caucasians (average ± standard deviation). Supplementary figure 1: The hip and knee moment during isolated isometric contraction of the hip-spanning muscle groups across a physiological range of motion. Supplementary figure 2: The calculated muscles force during isolated isometric contraction of the hip-spanning muscle groups across a physiological range of motion. Supplementary figure 3: The calculated hip force magnitude during isolated isometric contraction of the hip-spanning muscle groups across a physiological range of motion. Supplementary figure 4: The calculated tensile strain maps (top view) calculated using the extreme joint angles studied. [file 2873789.f1.zip › suppl_fig3_CMMM_1878445.docx]

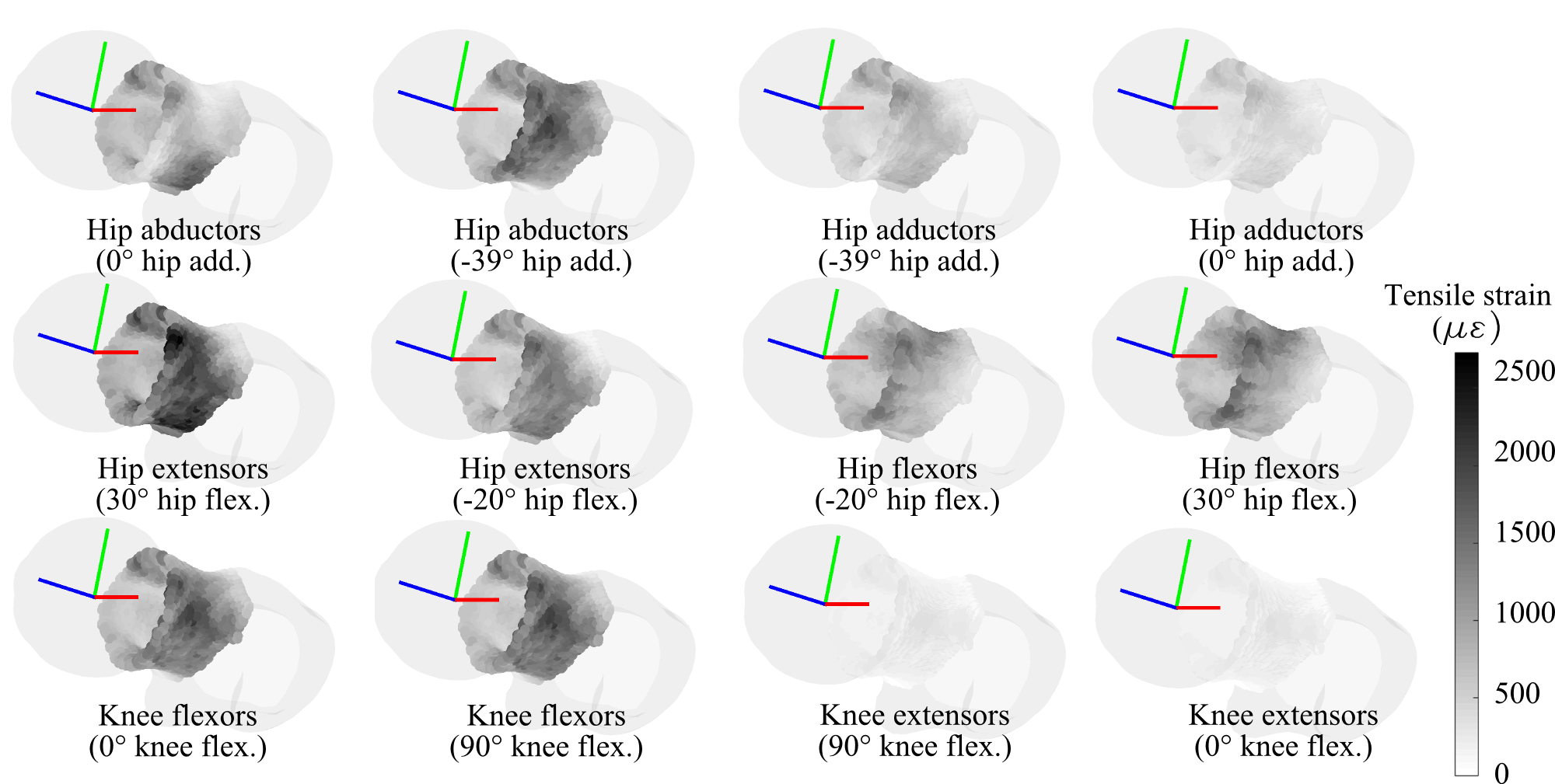

Supplement: Supplementary file 1 — Supplementary table 1: Comparison between salient geometrical parameters in the model and in adult Caucasians (average ± standard deviation). Supplementary figure 1: The hip and knee moment during isolated isometric contraction of the hip-spanning muscle groups across a physiological range of motion. Supplementary figure 2: The calculated muscles force during isolated isometric contraction of the hip-spanning muscle groups across a physiological range of motion. Supplementary figure 3: The calculated hip force magnitude during isolated isometric contraction of the hip-spanning muscle groups across a physiological range of motion. Supplementary figure 4: The calculated tensile strain maps (top view) calculated using the extreme joint angles studied. [file 2873789.f1.zip › suppl_fig4_CMMM_1878446.docx]
